# Théophile Alajouanine (1890–1980)

**DOI:** 10.1007/s00415-025-13240-8

**Published:** 2025-07-09

**Authors:** Bruno Kusznir Vitturi

**Affiliations:** Department of Neurology, University Hospital Schleswig-Holstein, Kiel, Germany

**Fig. 1:**
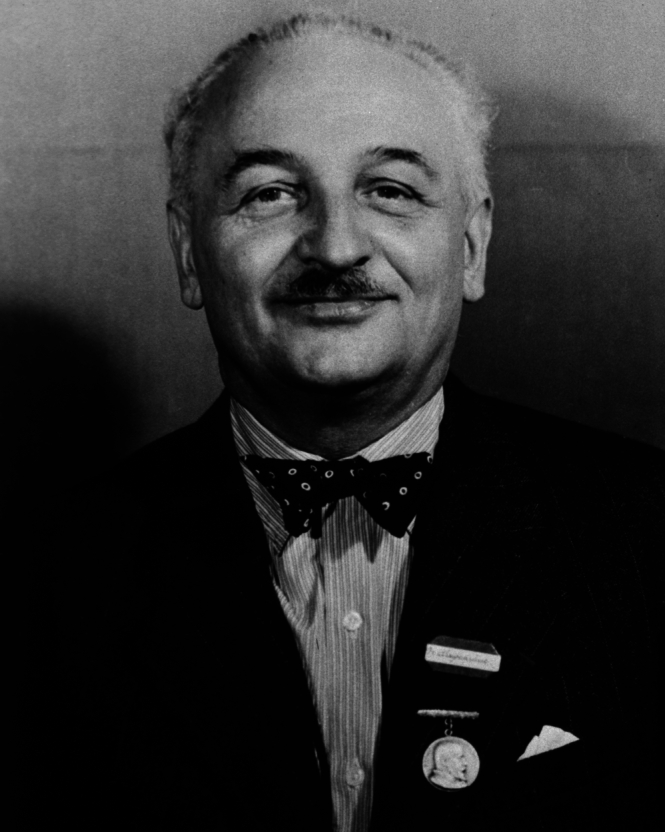
Théophile Alajouanine. Photo taken by neuropathologist Webb E. Haymaker (1902–1984) during the Fourth International Neurological Congress in Paris, 1949. Credit: Images from the History of Medicine (ID 101409194), National Library of Medicine, Bethesda, Maryland, USA

Born on June 12, 1890, in Verneix, France, Théophile A. J. Alajouanine (Fig. 1) initially found himself divided between two passions: art and medicine. Ultimately, he chose the medical path, inspired by the legacy of generations of neurologists whose discoveries had shaped modern clinical neuroscience. A gifted student, his academic brilliance led him into service during the First World War, after which he commenced his neurological training. His early education in psychiatry was soon enriched by the mentorship of notable figures such as Georges Guillain, Pierre Marie, and Charles Foix [1].

Alajouanine’s contributions to neurology are vast, but his most enduring legacy lies in the study of aphasia (e.g., *Le syndrome de désintégration phonétique dans l’aphasie*, Masson, 1939, with André Ombredane and Marguerite Durand; *L’aphasie et la désintégration fonctionnelle du langage*, Expansion Scientifique Française, 1944, with Pierre Mozziconacci; *L’aphasie et le langage pathologique*, Baillière, 1968). He was among the first to conceptualize language through a dual-system model, distinguishing between a sensory-motor component and a semantic system responsible for connecting language to thought [1]. His research extended far beyond Broca’s aphasia, addressing complex phenomena like stereotyped speech, agrammatism, word-finding deficits, aphasia in children, deafness, polyglots, artists, left-handed individuals, and even the introspective abilities of aphasic patients. He also worked extensively on speech therapy approaches and the psychological state of patients affected by language disorders [1].

One of Alajouanine's key neuropathological contributions emerged from a collaboration with Foix in 1926. Based on autopsy data, they described a spinal cord condition characterized by hypertrophy of the arterial media and intima, with marked fibrotic changes and degradation of the internal elastic lamina—a condition they termed “endo-meso-vasculitis” [2, 3]. The disorder (“Foix–Alajouanine syndrome”), later understood as spinal dural arteriovenous fistula (AVF), often presents with thoracolumbar paraparesis, sensory loss in the lower limbs, and bowel, bladder, and sexual dysfunction, typically following a relapsing–remitting course. Today, diagnosis relies on MRI findings that reveal intradural “flow voids”, indicative of these vascular malformations [2, 3].

In 1931, alongside the Brazilian neurologist Abraham Akerman, Alajouanine described a peculiar motor sign in a multiple sclerosis (MS) patient—later named the “unstable ataxic hand” [4]. The phenomenon was observed when the patient extended her arms and fingers (the “oath sign”), during which the hand failed to remain steady, with each finger moving independently. This condition later became known as “useless hand syndrome,” associated with cervical spinal MS. Charles Poser, a pioneer in the development of MS diagnostic criteria, considered this motor sign pathognomonic of the disease [4].

Alajouanine’s affinity for the arts deeply influenced his scientific outlook, particularly in his investigations into the intersection of neurology and artistic expression. In 1948, he documented the post-stroke aphasia of the composer Maurice Ravel, reflecting on how language impairment abruptly ended the composer’s creative output [5]. Similarly, he studied the painter Paul-Elie Gernez, who, despite suffering a stroke, continued producing artwork of undiminished quality. Alajouanine noted: “Gernez’s artistic production was just as perfect, and he did not experience any change in his artistic skills or style” [6]. Alajouanine’s fascination with the preservation of artistic abilities in aphasic individuals revealed his sensitivity to the resilience of the creative mind amid neurological adversity [7]. Alajouanine dedicated an article, published in *Brain*, in which he analyzed and interpreted Dostoevsky’s epilepsy and its influence on the Russian writer’s creative output [8]. Later, stroke would also affect other renowned individuals, rendering them aphasic—though not silent—thus reinforcing Alajouanine’s view that aphasia is not incompatible with artistic expression [9].

In addition to his work on language and neurovascular disorders, Alajouanine was also the first to describe the pathology of nucleus pulposus herniation. He proposed that discectomy could serve as an effective treatment in certain cases. This theory was put into practice in 1928, when his colleague and surgeon Daniel Petit-Dutaillis performed the first such operation on one of Alajouanine’s patients [10].

Alajouanine is eponymously remembered in the “Marie–Foix–Alajouanine syndrome”, an atrophy of the cerebellar cortex and ataxia at advanced age often associated with alcohol abuse, as well as the “Alajouanine maneuver”, i.e., compensatory eye movements when the position of the head changes.

Regarded as a student of Joseph-Jules Dejerine, Alajouanine reached the pinnacle of his academic career in 1947 when he was appointed Chair of Neurology, becoming the fifth successor to Jean-Martin Charcot at La Salpêtrière. Those close to the “pope of aphasia”, described him as a man of few words. According to Lhermitte, Lecours, and Signoret, during clinical presentations, “he often paused mid-sentence to roll a cigarette with black tobacco”—moments in which he was silently contemplating his next insight [10].

Théophile Alajouanine died in 1980, leaving behind a legacy that bridged neurology and the humanities. His life’s work serves as a testament to the idea that scientific inquiry and artistic sensibility may not only coexist but also mutually illuminate the human condition.
